# Smoking during Pregnancy Is Associated with a Decreased Incidence of Obstetric Anal Sphincter Injuries in Nulliparous Women

**DOI:** 10.1371/journal.pone.0041014

**Published:** 2012-07-16

**Authors:** Sari Räisänen, Katri Vehviläinen-Julkunen, Mika Gissler, Seppo Heinonen

**Affiliations:** 1 Department of Nursing, Savonia University of Applied Sciences, Iisalmi, Finland; 2 Department of Nursing Science, University of Eastern Finland, Kuopio, Finland; 3 Research Unit, Kuopio University Hospital, Kuopio, Finland; 4 Information Department, Welfare and Health Data Resources Unit, National Institute for Health and Welfare, Helsinki, Finland; 5 Nordic School of Public Health, Gothenburg, Sweden; 6 Department of Obstetrics and Gynecology, Kuopio University Hospital, Kuopio, Finland; 7 Institute of Clinical Medicine, University of Eastern Finland, Kuopio, Finland; John Hopkins Bloomberg School of Public Health, United States of America

## Abstract

**Background:**

Smoking is a modifiable lifestyle factor that has been shown to be associated with adverse perinatal outcomes and to have adverse health and dose-dependent connective tissue effects. The objective of this study was to examine whether smoking during pregnancy was associated with the incidence of obstetric anal sphincter injuries (OASIS) among six birthweight groups in singleton vaginal deliveries, considering nulliparous and multiparous women separately between 1997 and 2007 in Finland.

**Methodology:**

A retrospective population-based register study. Populations included women with spontaneous singleton vaginal deliveries, consisting of all 213,059 nulliparous and all 288,391 multiparous women. Incidence of OASIS (n = 2,787) between smoking status groups was adjusted using logistic regression analyses.

**Principal Findings:**

Of the nulliparous women, 13.1% were smokers, 3.6% had given up smoking during the first trimester of their pregnancy and 81.1% were non-smokers. Among these groups 0.7%, 0.9% and 1.1%, respectively suffered OASIS (p≤0.001). Nulliparous women who smoked had a 28% (95% CI 16–38%, p≤0.001) lower risk of OASIS compared to non-smokers, when adjusting for background variables. In multiparous women, the overall frequencies of OASIS were much lower (0.0–0.2%). A similar inverse relationship between OASIS rates and smoking was significant in pooled univariate analysis of multiparous women, but multivariate analysis revealed statistically insignificant results between non-smokers and smokers.

**Conclusions:**

Nulliparous women who were smokers had a 28% lower incidence of OASIS. However, smoking during pregnancy cannot be recommended since it has shown to be associated with other adverse pregnancy outcomes and adverse health effects. The observed association warrants clinical repetition studies and, if confirmed, also *in vitro* studies focusing on connective tissue properties at a molecular and cellular level.

## Introduction

Birth injury has been chosen as one of the indicators of patient safety and quality of care by the Organization for Economic Cooperation and Development (OECD) and Nordic countries [1], [2]. Obstetric anal sphincter injury (OASIS) is a serious complication of vaginal delivery that encompasses both third- and fourth-degree perineal ruptures [3] that result in anal incontinence in ca. 30–60% of those affected [4]. Incidence of OASIS varies substantially between countries and over time [5], [6]. The anal sphincter can be damaged in three ways during childbirth: a mechanical or traumatic injury, a neurological injury or a combined mechanical and neurological trauma [7]. The risk of mechanical trauma is greatest during a first vaginal delivery especially in operative vaginal deliveries, because the tissue has to stretch to accommodate the increasing diameter of the infant’s head much more rapidly than it would during a spontaneous delivery [8]. Consequently, a high birthweight implies a higher risk of OASIS whereas performing episiotomy, where the vaginal opening is enlarged for birth, reduces the stretching of the tissue and lowers the risk of severe perineal tears [9]. In previous studies focusing on OASIS, the role of obstetric risk factors in epidemiology and etiology have been well established but very little emphasis has been placed on lifestyle factors.

**Table 1:** Mean of characteristics and distribution of interventions for nulliparous women delivering vaginally grouped by infant’s birthweight according to smoking status group between 1997 and 2007 in Finland (n = 217,778) (Chi-Square, Kruskal-Wallis tests).

Characteristic/intervention	<3,000 g	3,000–3,499 g	3,500–3,999 g	4,000–4,499 g	4,500–4,999 g	≥5,000 g	Pooled
	% or mean	% or mean	% or mean	% or mean	% or mean	% or mean	% or mean
**OASIS, n (%)**							
Smoking	10 (0.2)**	61 (0.5)**	80 (0.9)**	39 (1.7)	0**	0	190 (0.7)***
Given up smoking	5 (0.5)	21 (0.8)	34 (1.2)	12 (1.2)	0	0	72 (0.9)
Non-smoking	119 (0.5)	553 (0.9)	839 (1.3)	421 (2.0)	73 (2.8)	4 (3.2)	2009 (1.1)
**Mean maternal age**							
Smoking	24.8***	24.4***	24.3***	24.2***	27.5***	23.9*	24.4***
Given up smoking	25.4	25.0	24.9	24. 8	24.7	23.8	25.0
Non-smoking	28.0	27.6	27.5	27.4	23.7	27.0	27.6
**Mean maternal weight (kg)**							
Smoking	61.5**	63.8	67.4***	69.0	71.4	81.7	64.8*
Given up smoking	61.6	63.1	67.1	69.2	73.3	87.8	65.3
Non-smoking	62.0	63.3	65.9	68.8	72.3	75.1	64.8
**Mean birthweight (g)**							
Smoking	2653.0***	3258.0***	3707.4***	4172.4	4629.2	5098.1	3345.5***
Given up smoking	2656.6	3270.8	3724.0	4181.7	4636.2	5157.3	3490.9
Non-smoking	2669.1	3271.7	3719.1	4175.6	4646.4	5151.9	3475.5
**Mean birth height (cm)**							
Smoking	46.8***	49.4***	50.9***	52.3***	53.6**	53.8	49.5***
Given up smoking	46.9	49.5	51.0	52.4	53.6	54.5	50.1
Non-smoking	47.1	49.6	51.1	52.5	53.8	55.1	50.2
**Mean head circumference (cm)**							
Smoking	33.0	34.2***	35.2**	36.0	36.7	38.0*	34.4***
Given up smoking	32.9	34.3	35.3	36.0	36.9	39.0	34.7
Non-smoking	33.0	34.4	35.3	36.1	36.9	37.1	34.7
**Vacuum**							
Smoking	9.5**	11.7***	14.8***	18.7	20.5	0	12.8***
Given up smoking	9.5	13.0	15.9	21.3	28.5	23.0	14.9
Non-smoking	11.0	13.8	16.9	20.6	23.7	31.2	12.8
**Mean length of active 2^nd^ stage (min.)**							
Smoking	26.9***	34.3***	39.8***	42.7***	54.7*	32.3	35.1***
Given up smoking	27.6	32.6	42.4	45.6	47.5	40.0	37.4
Non-smoking	32.8	41.0	48.1	51.0	65.3	55.0	44.3

* p value <0.05,

** p<0.01,

*** p<0.001.

Information on maternal height and weight, head circumference and length of the active second stage of birth have been recorded in the MBR since 2004 (n = 80,504).

Smoking is a modifiable lifestyle factor that has been shown to increase the risk of adverse pregnancy outcomes such as birth defects and restricted growth [10]. Smoking has also been shown to have independent, dose-dependent connective tissue effects such as premature skin aging [11], bone loss that increases the fracture risk in clinical settings [12], [13], poor periodontal conditions [14] and effects on growth factors and collagen synthesis both *in vivo* and *in vitro* [15], [16]. Further, there were several studies that revealed connections between smoking, the etiology of pelvic organ prolapse (POP) and urinary incontinence (UI). Childbirth injury is the main risk factor associated with POP and UI, increasing parity being associated with a greater risk of prolapse [17], [18]. It has been suggested, in turn, that cigarette smoking is associated with the development of POP and stress urinary incontinence (SUI) due to chronic coughing, increased abdominal pressure and inhaled chemical compounds. However, previous studies have not conclusively demonstrated such a relationship nor have they produced consistent results and, interestingly, one Norwegian study demonstrated that smoking is associated with SUI but not with severe SUI [19], [20]. Furthermore, a retrospective cohort study from the USA (n = 149,554) did not find a significant association between smoking, POP and UI and other related conditions [21].

Collectively, these results might imply that smoking may change the nature of the childbirth injury. We found several studies that reported an association between smoking and OASIS with inconsistent results. One large population study suggested a 40% (unadjusted OR 0.6, 95% CI 0.6–07) lower risk of OASIS among women who smoked at the end of the pregnancy (n = 28,566 of 266,037) [22] whereas the other studies that examined considerably lower numbers of cases found no significant associations [23]–[26]. This study was undertaken to evaluate whether smoking during pregnancy was associated with OASIS, one of the markers of childbirth injuries.

**Table 2:** Mean of characteristics and distribution of interventions for multiparous women delivering vaginally grouped by infant’s birthweight according to smoking status group between 1997 and 2007 in Finland (n = 296,963) (Chi-Square, Kruskal-Wallis tests).

Characteristic/intervention	<3,000 g	3,000–3,499 g	3,500–3,999 g	4,000–4,499 g	4,500–4,999 g	≥5,000 g	Pooled
	% or mean	% or mean	% or mean	% or mean	% or mean	% or mean	% or mean
**OASIS, n (%)**							
Smoking	4 (0.1)	7 (0.2)	20 (0.2)	12 (0.3)	3 (0.5)	0	46 (0.1)**
Given up smoking	0	1 (0.1)	0	1 (0.1)	0	0	2 (0.0)
Non-smoking	9 (0.0)	56 (0.1)	192 (0.2)	155 (0.3)	48 (0.5)	8 (0.9)	468 (0.2)
**Mean maternal age**							
Smoking	29.6***	29.1***	29.2***	29.5***	29.8***	31.4	29.3***
Given up smoking	29.7	29.3	29.5	29.5	29.0	31.7	29.4
Non-smoking	31.1	30.9	31.0	31.1	31.4	31.5	31.0
**Mean maternal weight (kg)**							
Smoking	62.7	66.0***	69.3***	74.0***	78.3***	82.7	67.6***
Given up smoking	63.6	65.4	68.3	71.0	75.0	77.2	67.7
Non-smoking	62.7	64.4	67.0	70.4	73.3	78.5	66.8
**Mean birthweight (g)**							
Smoking	2642.4***	3264.5***	3720.3***	4181.5***	4651.7	5201.0	3443.2***
Given up smoking	2683.1	3285.0	3740.5	4195.7	4661.4	5120.4	3630.2
Non-smoking	2659.8	3287.4	3737.9	4193.0	4659.4	5175.2	3665.9
**Mean birth height (cm)**							
Smoking	46.6***	49.2***	50.7***	52.1***	53.4**	54.6	49.7***
Given up smoking	46.9	49.4	50.8	52.1	53.4	54.7	50.4
Non-smoking	46.9	49.5	50.9	52.3	53.6	54.9	50.6
**Mean head circumference (cm)**							
Smoking	33.0	34.4**	35.3**	36.2	37.0	37.6	34.7***
Given up smoking	33.2	34.3	35.3	36.1	36.9	37.6	35.0
Non-smoking	33.0	34.4	35.3	36.2	36.9	37.6	35.1
**Vacuum**							
Smoking	1.5	1.7	2.3	3.0	3.6	2.0	2.0
Given up smoking	0.7	2.3	2.4	3.3	1.9	0	2.3
Non-smoking	1.6	1.7	2.1	2.7	3.4	4.4	2.2
**Mean length of active 2^nd^ stage (min.)**							
Smoking	9.4	11.0	13.2	14.2	18.6	36.7	12.0***
Given up smoking	9.0	11.9	14.2	16.2	20.1	25.2	13.6
Non-smoking	9.7	11.4	13.6	15.4	18.1	23.5	13.2

* p value <0.05,

** p<0.01,

*** p<0.001.

Information on maternal height and weight, head circumference and length of the active second stage of birth have been recorded in the MBR since 2004 (n = 80,504).

## Methods

### Objectives

We have previously determined risk profiles of OASIS separately among nulliparous and multiparous women using the same database as used in this study [27], [28]. The purpose of this study was to examine whether smoking during pregnancy was associated with the incidence of OASIS, as originally suggested by the unadjusted results of Baghestan et al. [22]. Notably, smoking results in lower birthweights [10], [29]: high birthweight is one of the risk factors for OASIS [22], [28]. Therefore, we analyzed the data for six birthweight categories of singleton vaginal deliveries, considering nulliparous and multiparous women separately; the data were for the period 1997–2007 in Finland and were further adjusted to account for background factors.

The source of data for this study was the Medical Birth Register (MBR), which includes information on maternal and neonatal birth characteristics and perinatal outcomes (all live births or stillbirths at 22 gestational weeks or later, or weighing 500 g or more) for all women who have given birth in Finland and for their newborn infants. The data for the period 1997–2007 are derived from the clinical records of all obstetric care units in Finland; the register is currently maintained by the National Institute for Health and Welfare.

**Figure 1:**
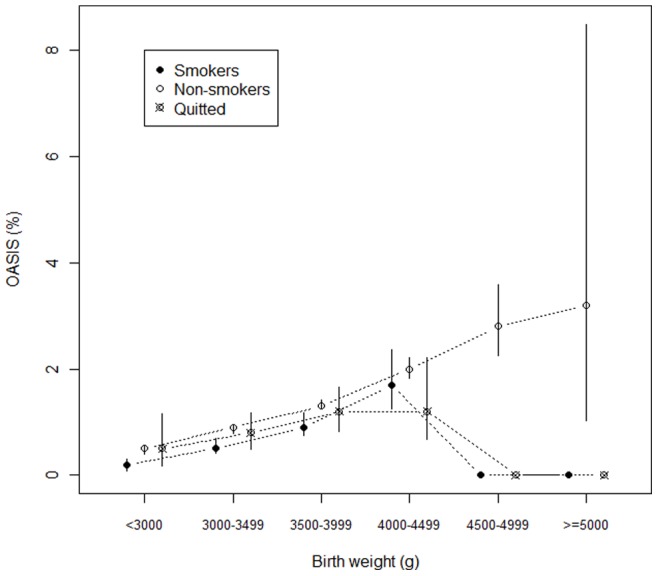
Obstetric anal sphincter injury rates (95% CIs) according to smoking status group among six birthweight categories in nulliparae.

Information on OASIS has been recorded in the MBR since 2004. For the years 1997 to 2003, the information was taken from the Hospital Discharge Register (HDR), based on ICD-10 codes O70.2 (3^rd^ degree) and O70.3 (4^th^ degree). We also had information concerning all aspects of care during pregnancy and birth, such as medical interventions and surgical procedures. The two data sources were linked together via encrypted unique personal identification numbers.

The degree of OASIS was classified according to standard definitions: a third degree rupture involves the external anal sphincter and a fourth degree rupture affects both the anal sphincter and anorectal mucosa [3]. Information on smoking habits was based on data collected by self-administered paper or electronic questionnaire. However, we did not have additional information on smoking such as how long the patient had been a smoker or the number of cigarettes smoked each day.

**Table 3:** Adjusted risk of OASIS for nulliparous women delivering vaginally and nulliparous women grouped by birthweight between 1997 and 2007 in Finland (Logistic regression analyses).

Characteristic/outcome	Nulliparae	Nulliparae with <3000 g infant	Nulliparae with 3000–4000 g infant	Nulliparae with >4000 g infant
n	n = 213,059	n = 32,246	n = 153,644	n = 27,169
Nulliparous women	1	1	1	1
First vaginal delivery after CS	1.54 (1.35–1.77)	1.88 (1.00–3.52)	1.53 (1.29–1.81)	1.44 (1.13–1.85)
Mode of delivery				
Vaginal delivery	1	1	1	1
Breech	0.96 (0.52–1.80)	0.39 (0.05–2.80)	1.34 (0.56–2.28)	1.16 (0.16–8.80)
Forceps	4.87 (3.06–7.74)	12.60 (2.97–53.43)	6.17 (3.65–10.41)	1.44 (0.36–6.00)
Vacuum assistance	3.00 (2.72–3.29)	3.07 (2.05–4.60)	3.31 (2.96–3.70)	2.27 (1.89–2.74)
Maternal age (year)				
≤19	1	1	1	1
20–29	1.56 (1.22–1.99)	2.22 (0.70–7.13)	1.44 (1.09–1.91)	1.88 (1.10–3.34)
30–39	1.95 (1.52–2.50)	2.46 (0.76–8.01)	1.77 (1.33–2.37)	2.51 (1.44–4.36)
≥40	1.51 (0.97–2.36)	0.86 (0.09–8.40)	1.49 (0.90–2.47)	1.73(0.61–4.87)
Birth weight (g)				
<3000	1	–	–	–
3000–4000	2.35 (1.97–2.81)			
>4000	4.30 (3.55–5.21)			
1Length of active 2^nd^ stage (min)				
≤15	1	1	1	1
16–30	1.39 (1.10–1.73)	2.53 (1.19–5.36)	1.33 (1.02–1.73)	1.26 (0.75–2.11)
31–45	1.82 (1.45–2.30)	2.19 (0.90–5.32)	1.82 (1.39–2.39)	1.67 (1.00–2.78)
46–60	2.10 (1.64–2.69)	1.31 (0.36–4.83)	1.97 (1.47–2.65)	2.41 (1.43–4.06)
≥61	2.32 (1.85–2.89)	1.76 (0.62–5.02)	2.38 (1.83–3.09)	2.18 (1.34–3.56)
Episiotomy	0.73 (0.67–0.80)	1.06 (0.74–1.53)	0.75 (0.67–0.84)	0.60 (0.50–0.72)
Epidural analgesia	0.87 (0.80–0.95)	0.59 (0.42–0.83)	0.88 (0.80–0.97)	0.93 (0.78–1.11)
*Smoking status*				
Non-smoking	1	1	1	1
Given up smoking	0.87 (0.67–1.10)	1.09 (0.44–2.68)	0.97 (0.74–1.27)	0.57 (0.32–1.01)
Smoking	0.72 (0.62–0.84)	0.39 (0.20–0.74)	0.73 (0.61–0.87)	0.85 (0.61–1.19)

1 Length of active 2^nd^ stage of birth adjusted only for the years 2004–2007. CS = Cesarean section.

### Participants

Data for nulliparous (n = 213,059) and multiparous women (n = 288,391) were analyzed separately because the former are known to be at a far greater risk of OASIS than the latter [22]. Women who were admitted for vaginal delivery after a previous caesarean section for their first birth were classified as nulliparous (27,819, 12.8% of all nulliparous women).

### Ethics

A statement from the local ethics committee was not needed since no human experimentation was conducted. The data used in our study was based on health register data from the National Institute for Health and Welfare. The register keeper gave the necessary authorization for the use of their sensitive health register data in scientific research, as required by national data protection legislation. The Data Protection Authority was informed about the study, as required by national data protection legislation. No informed consent by the individuals on the register was needed, since the study was completely based on anonymized information and no person on the register was contacted.

### Statistical Methods

The Chi-Square and Kruskal-Wallis tests were used, respectively, to assess the differences in OASIS between smoking status groups and categorical classifications (OASIS and vacuum), or continuous variables that were not normally distributed (maternal age, maternal weight, birthweight and height, head circumference and length of active second stage of birth), among the six birthweight groups. Birthweight was divided into six categories: less than 3000, 3000–3499, 3500–3999, 4000–4499, 4500–4999 and 5000 grams or more in the univariate analyses. Furthermore, the incidence of OASIS was adjusted for significant (*p<0.1*) and clinically important variables (maternal age, birthweight, the mode of delivery, episiotomy, epidural analgesia, length of active second stage of birth) and smoking status using logistic regression analysis. In the multivariate analyses, the birthweight was divided into three categories (less than 3000, 3000–3999 and greater than 4000 grams). The mode of delivery was recorded as vaginal spontaneous, breech, forceps or vacuum assisted. The type of episiotomy is exclusively lateral in Finland [30]. Information on maternal weight, head circumference, and length of the active second stage of birth has been recorded in the MBR since 2004 and thus we adjusted only for the years 2004 to 2007. The active second stage of birth was defined as the active phase of bearing down until the delivery of the infant. Smoking status was recorded as smoker, gave up smoking during the first trimester of pregnancy, or non-smoker. The rate of missing data for smoking status was 2.1% (n = 11,052) and these cases were removed prior to analysis. Differences were deemed to be significant if *p*<0.05. In all of the analyses, data on third and fourth degree obstetric anal sphincter ruptures were pooled. The data were analyzed using SPSS for Windows 19.0, Chicago, IL.

## Results

The data described 213,059 nulliparous and 288,391 multiparous women, of whom 2,271 (1.1%) and 516 (0.2%), respectively, suffered OASIS. There were significant differences in OASIS rates between the six birthweight categories among both groups of women (p≤0.001): the proportions of OASIS among the six birthweight categories increased with increasing birthweight in both groups of women, as shown in Tables 1 and 2.

Of the nulliparous women, 13.1% were smokers, 3.6% had given up smoking during pregnancy and 81.1% were non-smokers. Among these groups, 0.7%, 0.9% and 1.1%, respectively, suffered OASIS (p≤0.001). Table 1 and Figure 1 show that nulliparous women who were smokers or who had given up smoking experienced fewer OASIS than non-smokers in all birthweight categories, although only four of the differences between the groups were statistically significant. In nulliparous women, there were also significant differences in the mean maternal age, the mean length of the active second stage of birth and the proportion of vacuum assisted deliveries between smoking status groups (see Table 1). Multivariate analysis confirmed the results of the univariate analyses and, indeed, the incidence of OASIS was 28% lower (adjusted OR 0.72, 95% CI 0.62–0.84, p≤0.001) in nulliparous women who smoked compared to non-smokers (Table 3). Differences in the incidence of OASIS between nulliparous women who gave up smoking and non-smokers were not statistically significant. We also adjusted the incidence of OASIS among three birthweight groups (less than 3000, 3000–3999 and greater than 4000 grams) that somewhat confirmed the results of analysis performed among all the nulliparous women (Table 3). However, in women who gave birth to a child under 3000 grams, smoking during pregnancy was associated with a 61% lower risk (adjusted OR 0.39, 95% CI 0.20–0.74) of OASIS compared to non-smokers whereas results for women who gave birth to a child over 4000 grams were insignificant.

**Figure 2:**
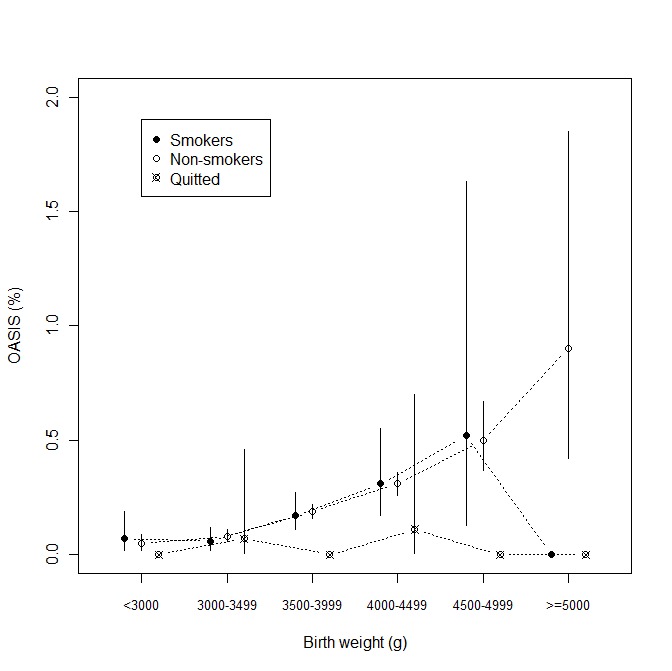
Obstetric anal sphincter injury rates (95% CIs) according to smoking status group among six birthweight categories in multiparae.

Of the multiparous women, 11.9% were smokers, of whom 0.1% had OASIS; less than 0.01% of the women who gave up smoking during the first trimester of pregnancy (1.7%) had OASIS, compared to an OASIS rate of 0.2% among non-smokers (p = 0.006). Figure 2 shows OASIS rates in multiparous women grouped by smoking status in the six birthweight categories. All these differences were statistically insignificant, as shown in Table 2. However, an inverse association between OASIS rates and smoking was significant in pooled univariate analysis among multiparous women. The results of multivariate analysis demonstrated statistically significant differences in the incidence of OASIS between non-smokers and women who had given up smoking during pregnancy (adjusted OR 0.23, 95% CI 0.06–0.91) (Table 4). However, the results remained insignificant in multivariate analyses performed on birthweight groups.

**Table 4:** Adjusted risk of OASIS for multiparous women delivering vaginally and multiparous women grouped by birthweight between 1997 and 2007 in Finland (Logistic regression analyses).

Characteristic/outcome	Multiparae	Multiparae with <3000 g infant	Multiparae with 3000–4000 g infant	Multiparae with >4000 g infant
N	n = 288,391	n = 25,184	n = 196,593	n = 66,614
Mode of delivery				
Vaginal delivery	1	1	1	1
Breech	0.79 (0.20–3.17)	–	1.02 (0.25–4.12)	–
Forceps	10.48 (2.53–43.35)	–	23.34 (5.55–98.14)	–
Vacuum assistance	4.23 (3.21–5.58)	6.50 (0.76–55.37)	4.29 (2.92–6.30)	4.14 (2.78–6.17)
Maternal age (year)				
≥29	1	1	1	1
30–39	1.25 (1.03–1.50)	0.30 (0.08–1.15)	1.43 (1.10–1.85)	1.14 (0.86–1.51)
≥40	1.52 (1.04–2.24)	1.83 (0.38–8.76)	1.65 (0.96–2.81)	1.33 (0.74–2.41)
Birth weight (g)				
<3000	1	–	–	–
3000–4000	2.59 (1.48–4.51)			
>4000	5.73 (3.27–10.05)			
1Length of active 2^nd^ stage (min)				
≤15	1	1	1	1
16–30	2.96 (2.11–4.14)	1.39 (0.17–11.43)	3.23 (2.09–5.26)	2.72 (1.63–4.54)
31–45	4.33 (2.78–6.74)	–	5.32 (2.94–9.62)	3.46 (1.75–6.84)
46–60	3.03 (1.48–6.20)	–	2.31 (0.70–7.64)	3.75 (1.50–9.40)
≥61	7.24 (4.37–12.00)	–	10.08 (5.24–19.37)	5.29 (2.38–11.77)
Episiotomy	2.04 (1.68–2.48)	0.45 (0.05–3.73)	2.41 (1.85–3.15)	1.75 (1.31–2.35)
Epidural analgesia	1.50 (1.24–1.82)	1.70 (0.52–5.58)	1.44 (1.10–1.87)	1.57 (1.18–2.08)
*Smoking status*				
Non-smoking	1	1	1	1
Given up smoking	0.23 (0.06–0.91)	–	0.21 (0.03–1.50)	0.27 (0.04–1.90)
Smoking	0.87 (0.64–1.18)	1.30 (0.40–4.23)	0.80 (0.54–1.19)	0.94 (0.55–1.58)

1 Length of active 2^nd^ stage of birth adjusted only for 2004–2007.

## Discussion

The aim of this study was to examine whether smoking during pregnancy was associated with the incidence of OASIS among six birthweight groups for singleton vaginal deliveries. The result was that nulliparous women who smoked during pregnancy had a 28% (95% CI 16–38%) lower incidence of OASIS than non-smokers, the difference being significant in both univariate and multivariate models. In multiparous women, the overall frequency of OASIS was one fifth of that observed in nulliparous women, but still a similar inverse relationship between smoking and OASIS was observed in pooled univariate analysis. This association, however, appeared to be insignificant in multivariate analyses between non-smokers and smokers but multiparous women who had given up smoking had a 77% (95% CI 9–94%) lower incidence of OASIS compared to non-smokers. For multiparous women, lack of statistical power hampered the comparisons of this subset of patients.

The present study provided new information on the association between smoking and OASIS rates. The results of the univariate analyses demonstrated that there was a lower incidence of OASIS in nulliparous women who were smokers or who had given up smoking during pregnancy compared to non-smokers. It was possible that the association between lower OASIS rates and smoking could have been explained by lower birthweight; in this study, however, the results did not change although the data were analyzed separately for six birthweight categories. Tables 1 and 2 show that there were no smokers among women with infants over 4,500 grams among the nulliparae and multiparae, respectively, that might have explained the insignificant results in the univariate analyses. The results of the multivariate analysis confirmed that nulliparous women who smoked had a 28% lower incidence of OASIS compared to non-smokers. Multivariate analyses performed separately for three birthweight groups showed that the relative risk of OASIS in smokers increased along with birthweight. This may reflect the fact that smoking during pregnancy is known to be associated with impaired fetal growth in an inverse dose-effect relationship [10]. In other words, women having high birthweight infants were likely to smoke less than those with low or normal birthweight infants and therefore the effect of smoking might have been less obvious in this high birthweight group. In multiparous women, the results were statistically insignificant between non-smokers and smokers, possibly due to the fact that OASIS among multiparae is very rare. Further, the lower incidence of OASIS in women who had given up smoking during pregnancy compared to smokers might suggest that some smokers had classified themselves as having given up smoking on the self-reported questionnaire.

Overall, the obstetric risk profile of OASIS was in line with previous results [22], [31]. Use of episiotomy and epidural anaesthesia had a complex association, each being associated with a reduced risk for nulliparae, but increased risk for multiparae suggesting confounding by indication, more complicated delivery and an already higher risk of OASIS [27].

The results of the present study were in line with a large population-based study from Norway pertaining to the risk factors of OASIS. They found that the incidence of OASIS was reduced by 40% among women who smoked at the end of the pregnancy [22]. Other studies with a lower number of cases or a different study setting and differences in obstetric practices, such as using a midline rather than a medio-lateral or lateral episiotomy as an intervention, showed no significant association between smoking and the risk of OASIS [5]–[8]. Based on the present results, an association between the lower incidence of OASIS and smoking during pregnancy is unexplained and the mechanism remains unclear. Further, we do not suggest that smoking during pregnancy reduces the risk of OASIS since it has shown to be associated with other adverse pregnancy outcomes such as birth defects, fetal hypoxia, restricted growth and adverse health effects such as cancer and stroke [10], [32]. The importance of these results is that they might suggest that smoking may interfere with collagen synthesis and connective tissue properties, thus modifying the OASIS risk by an, as yet, unknown mechanism [33].

In conclusion, the results showed that smoking during pregnancy was associated with a lower incidence of OASIS in nulliparae and the results concerning multiparae were in line with the results of nulliparae but remained partially insignificant possibly due to the low number of cases. However, based on the present study, the mechanism remains unclear; further research is needed to replicate these findings and to focus on the underlying biochemistry.

### Strengths and Weaknesses

The most important strength of this study was that the data were derived from the mandatory, national, population-based Medical Birth Register (MBR), which covers the entire population, has excellent coverage and contains good quality data [34], [35]. Nevertheless, it is possible that this kind of register information includes errors and missing values because the data are produced mainly for administrative and statistical purposes, not primarily for research. The data are always checked at the National Institute for Health and Welfare and returned for revision if necessary, thus increasing its quality. In addition, incidents that resulted in surgical repair and that have specific diagnosis codes, such as OASIS, are likely to be recorded correctly, since they are associated with extra costs for the procedure itself and usually result in prolonged hospital treatment. Also, it is possible that women failed to disclose their use of tobacco during pregnancy or they may have given up smoking during pregnancy but not during the first trimester, the only period that was recorded. In general, however, daily smoking during pregnancy is relatively well covered in the Finnish MBR [36]. Furthermore, the data did not provide information on how long a patient has been smoking for or the number of cigarettes smoked each day; therefore, we could not examine the cigarette smoking dose-response.

The information on OASIS has been recorded in the MBR since 2004 so the data for the years 1997 to 2003 were taken from Hospital Discharge Register (HDR). This register is also mandatory and its completeness and quality are high [37]. For example, for 2006–2007, with independent recording of OASIS in the two registers, HDR covered 95% of OASIS cases recorded in the MBR.
